# Drilling dimension effects in early stages of osseointegration and implant stability in a canine model

**DOI:** 10.4317/medoral.20557

**Published:** 2015-04-10

**Authors:** Felipe-Eduardo Baires-Campos, Ryo Jimbo, Estevam-Augusto Bonfante, Maiolino-Thomaz Fonseca-Oliveira, Camila Moura, Darceny Zanetta-Barbosa, Paulo-Guilherme Coelho

**Affiliations:** 1 DDS, PhD. Division of Oral and Maxillofacial Surgery, Department of Oral Surgery, School of Dentistry, Federal University of Uberlândia, Uberlândia, MG, Brazil; 2 DDS, PhD Department of Prosthodontics, Faculty of Odontology, Malmö University, Malmö, Sweden; 3 DDS, PhD Department of Prosthodontics, University of Sao Paulo – Bauru College of Dentistry, Bauru, SP, Brazil; 4 DDS, PhD Department of Histology, Federal University of Triângulo Mineiro, Uberaba, MG, Brazil; 5 DDS, BS, MSMtE, MS, PhD Department of Biomaterials and Biomimetics, New York University, New York, NY, USA. Director for Research, Department of Periodontology and Implant Dentistry New York University College of Dentistry, New York, NY, USA. Division of Engineering, New York University Abu Dhabi, Abu Dhabi, United Arab Emirates

## Abstract

**Background:**

This study histologically evaluated two implant designs: a classic thread design versus another specifically designed for healing chamber formation placed with two drilling protocols.

**Material and Methods:**

Forty dental implants (4.1 mm diameter) with two different macrogeometries were inserted in the tibia of 10 Beagle dogs, and maximum insertion torque was recorded. Drilling techniques were: until 3.75 mm (regular-group); and until 4.0 mm diameter (overdrilling-group) for both implant designs. At 2 and 4 weeks, samples were retrieved and processed for histomorphometric analysis. For torque and BIC (bone-to-implant contact) and BAFO (bone area fraction occupied), a general-linear model was employed including instrumentation technique and time in vivo as independent.

**Results:**

The insertion torque recorded for each implant design and drilling group significantly decreased as a function of increasing drilling diameter for both implant designs (*p*<0.001). No significant differences were detected between implant designs for each drilling technique (*p*>0.18). A significant increase in BIC was observed from 2 to 4 weeks for both implants placed with the overdrilling technique (*p*<0.03) only, but not for those placed in the 3.75 mm drilling sites (*p*>0.32).

**Conclusions:**

Despite the differences between implant designs and drilling technique an intramembranous-like healing mode with newly formed woven bone prevailed.

**Key words:** Histomorphometry, biomechanical, in vivo, initial stability, insertion torque, osseointegration.

## Introduction

Osseointegration has been thoroughly reported in literature and has become one of the most effective treatments in medicine and dentistry. While highly successful for anchoring load bearing capable metallic devices through established surgical and prosthetic techniques, a plethora of scientific work regarding osseointegration’s fundamental mechanisms as a function of multiple variables has been published over the last decade (1).

Being the implant surface the first part of the implant to interact with the host, a large amount of work has established surface modifications as one possible design parameter capable of substantially decrease osseointegration time (2-4). For that purpose, surface texturing has become the most utilized approach and work regarding texturing at both micrometer scale and nanometer scale is ongoing in an attempt to find the optimal pattern to hasten early osseointegration (4-6). In addition, recent work on how the dimensional interplay between implant macro-geometry and surgical instrumentation affects the bone healing pathway has provided new insight regarding how implant systems can be further modified to provide scenarios where implant stability can be immediately achieved and temporally maximized (7-10).

While it has been previously demonstrated that surgical instrumentation and implant design may result in high degrees of contact between implant and bone immediately after placement providing higher resistance to micromotion (thus improving the likelihood of osseointegration compared to fibrous integration) (11), several studies have shown that this initially contacting bone gradually resorbs resulting in an implant stability dip (7,8,12). Such decrease in implant resistance to micromotion has been experimentally demonstrated to be posteriorly compensated by new bone formation at regions where bone resorption occurred (13-15). Such scenario has been suggested to arise from the excessive strain or compression that the implant exerts on the surrounding bone that exceeds the physiological limit and triggers bone resorption/remodeling (13).

Alternatively, void spaces left between bone and implant bulk that will fill with a blood clot immediately after placement and will not contribute to primary stability are known to rapidly fill with woven bone, being a key contributor to secondary stability as it does not have to undergo remodeling due to its different healing pattern (16,17). The mode and kinetics of bone formation in such healing chambers has been discussed in detail by Berglundh *et al*. (14) while the effect of healing chamber size and shape on bone formation has been explored by Marin *et al*. (9). In addition, studies comparing implants that were tightly fit into their drilling sites with implants that were simply taped into oversized drilling sites or lightly screwed in larger drilling sites have shown that at the same time that bone resorption was occurring in regions that were compressed by the implant, bone filling was already occurring in healing chambers (7,8,12,18). Altogether, these studies have led to an initial platform for designing implant systems that combine instrumentation and implant geometrical configurations that attempt to maximize implant stability over time. For this purpose, several investigators have employed either experimental implant designs with an outer thread design that provided stability while the inner thread and osteotomy dimensions allowed healing chambers (14,19,20) or alterations in osteotomy dimensions in large thread pitch implant designs (7,8,12). While it is obvious that most threaded implant systems may present healing chambers if expanded drilling dimensions are utilized, the combination of the initial mechanical stability and related healing may not necessarily lead to satisfactory degrees of atemporal stability. Thus, the objective of the present study was to histologically evaluate two different implant systems, a classic thread design versus another specifically designed for healing chamber formation along with primary stability, placed into two drilling schemes that allowed different initial stability and interplay between implant and bone.

## Material and Methods

Implants of 4.1 mm diameter and 10 mm length of two different macrogeometries (n=40 of each type) were utilized in the present study, namely Strong SW and Unitite (SIN, São Paulo, SP, Brazil). Both implants presented similar conical profiles and the main difference between the designs comprised the thread profile, where the SW presented a single thread design and the Unitite presented a dual thread (thread within thread profile). Both implants presented a dual acid etched surface that has been previously characterized (21). Two drilling techniques were utilized for each of the implant designs, a technique to the final diameter recommended by the manufacturer (3.75 mm- regular group) and a technique where the final diameter was larger than recommended by the manufacturer (4.0 mm- over drilling group). For the laboratory in vivo model, ten adult male beagle dogs with approximately 1.5 years of age were acquired following the approval of the Ethics Committee for Animal Research at “Institution’s name can not be mentioned according to author’s guidelines”, (protocol/approval number CEUA/UFU 082/12).

Prior to general anesthesia, IM atropine sulfate (0,044 mg/kg) and xilazyne chlorate (8 mg/kg) were administered. A 15mg/Kg ketamine chlorate dose was then utilized to achieve general anesthesia.

The surgical site was the proximal tibia. Following hair shaving, skin exposure, and antiseptic cleaning with iodine solution at the surgical and surrounding area, a 5 cm length incision to access the periosteum was performed and a flap reflected for bone exposure.

Four implants were placed along the tibia from proximal to distal in an alternated implant design and drilling technique distribution, being interchanged in every tibia to minimize bias from different implantation sites (sites 1 to 4 from proximal to distal). Therefore, the 40 implants of each design in each drilling technique remained in vivo for either 2 or 4 weeks, respectively, and were thus allocated in sites 1 to 4 in an equal distribution. This approach resulted in balanced surgical procedures that allowed the comparison of the same number of implant design and drilling technique per time in vivo, limb, surgical site (1 through 4), and animal. The implants were placed at distances of 1 cm from each other along the central region of the bone. The implants were inserted in the drilled sites and the maximum insertion torque was recorded with a portable digital torque meter (Tohnichi, Tokyo, Japan) with a 200 Ncm load cell for each implant placed.

Following placement, each implant received its proprietary cover screw to avoid tissue overgrowth. The soft tissue was sutured in layers following standard procedures, where the periosteum was sutured with vicryl 4-0 (Ethicon Johnson, Miami, FL, USA) and the skin with 4-0 nylon (Ethicon Johnson, Miami, FL, USA).

Postoperative antibiotic and anti-inflammatory medication included a single dose of Benzyl Penicillin Benzatine (20.000 UI/Kg) IM and Ketoprofen 1% (1ml/5Kg). The animals were euthanized by anesthesia overdose and the limbs were retrieved by sharp dissection. The soft tissue was removed by surgical blades, and initial clinical evaluation was performed to determine implant stability. If an implant was clinically unstable, it was excluded from the study.

The bones containing the implants were reduced to blocks and immersed in 10% buffered formalin solution for 24h. The blocks were then washed in running water for 24h, and steadily dehydrated in a series of alcohol solutions ranging from 70-100% ethanol. Following dehydration, the samples were embedded in a methacrylate-based resin (Technovit 9100, Heraeus Kulzer GmbH, Wehrheim, Germany) according to the manufacturer’s instructions. The blocks were then cut into slices (~300 µm thickness) aiming the center of the implant along its long axis with a precision diamond saw (Isomet 2000, Buehler Ltd., Lake Bluff, USA), glued to acrylic plates with an acrylate-based cement, and a 24h setting time was allowed prior to grinding and polishing. The sections were then reduced to a final thickness of ~30 µm by means of a series of SiC abrasive papers (400, 600, 800, 1200 and 2400) (Buehler Ltd., Lake Bluff, IL, USA) in a grinding/polishing machine (Metaserv 3000, Buehler Ltd., Lake Bluff, USA) under water irrigation (22). The sections were then toluidine blue stained and referred to optical microscopy at 50X-200X magnification (Leica DM2500M, Leica Microsystems GmbH, Wetzlar, Germany) for histomorphologic evaluation.

The bone-to-implant contact (BIC) was determined at 50X-200X magnification (Leica DM2500M, Leica Microsystems GmbH, Wetzlar, Germany) by means of computer software (Leica Application Suite, Leica Microsystems GmbH, Wetzlar, Germany). The regions of bone-to-implant contact along the implant perimeter were subtracted from the total implant perimeter, and calculations were performed to determine the BIC. The bone area fraction occupied (BAFO) between threads in trabecular bone regions was determined at 100X magnification (Leica DM2500M, Leica Microsystems GmbH, Wetzlar, Germany) by means of a computer software (Leica Application Suite, Leica Microsystems GmbH, Wetzlar, Germany). The areas occupied by bone were subtracted from the total area between threads, and calculations were performed to determine the BAFO (reported in percentage values of bone area fraction occupied) (16).

For all outcomes, statistical significance was set to 95% level of confidence and the number of dogs was considered as the statistical unit for all comparisons. For the torque and histomorphometric dependent variables BIC and BAFO, a general linear model was employed including implant design, instrumentation technique, and time in vivo as independent variables (surgical site position was preliminarily evaluated and due to a lack of effect on Torque, BIC, and BAFO was excluded from further analysis) (IBM SPSS Statistics, v. 19 IBM, New York, NY, USA).

## Results

No complications regarding procedural conditions or other immediate clinical concerns were observed during immediate follow up and throughout the entire study in vivo period. No postoperative complication was detected and no implant was excluded from the study due to clinical instability of all implants after euthanization.

**Figure 1:**
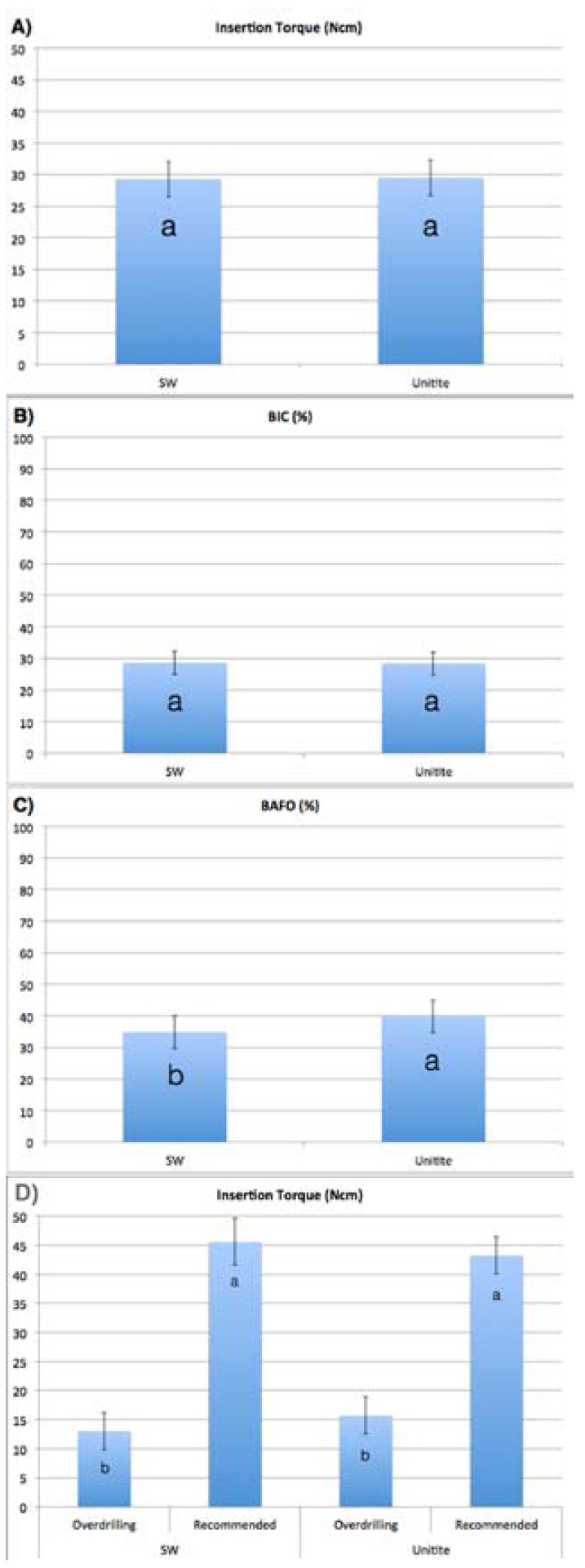
Overall (a) insertion torque, (b) BIC, and (c) BAFO for the two different implant designs. (d) Insertion torque values for the two different implant designs placed with the recommended and the over drilling instrumentation protocol. The error bars represent the 95% confidence interval and letters represent statistically homogeneous groups.

Overall torque (when both drilling techniques were collapsed for each separate implant design) showed no significant difference between implant designs (Fig. 1a). The insertion torque recorded for each implant design and drilling group is presented in fig. 1d, where the torque significantly decreased as a function of increasing drilling diameter for both implant designs (*p*<0.001). No significant differences were detected between implant designs for each drilling technique (*p*>0.18).

**Figure 2:**
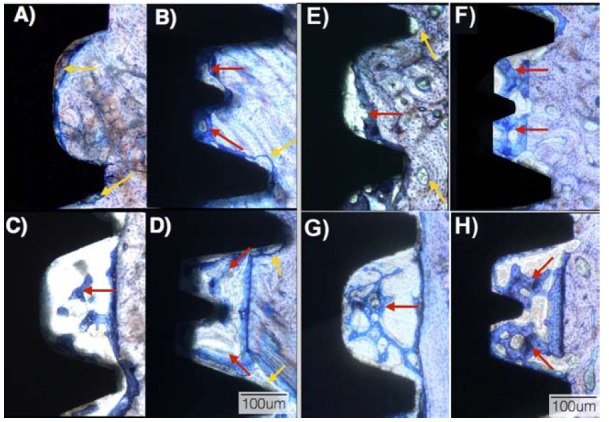
Optical micrographs at 2 weeks in vivo for the (a) SW recommended instrumentation, (b) Unitite recommended instrumentation, (c) SW overdrilling instrumentation, and (d) Unitite overdrilling instrumentation. Optical micrographs at 4 weeks in vivo for the (e) SW recommended instrumentation, (f) Unitite recommended instrumentation, (g) SW overdrilling instrumentation, and (h) Unitite overdrilling instrumentation. The red arrows depict newly formed bone at the healing chambers regions; yellow arrows depict bone remodeling regions.

Qualitative evaluation of the biological response showed intimate contact between cortical and trabecular bone for all groups at both implantation times, including regions that were in close proximity or substantially away from the osteotomy walls (Fig. 2). For both implant designs and all drilling techniques employed, the toluidine blue stained thin sections presented an interfacial remodeling bone healing mode at regions where intimate contact existed between implant surfaces and bone immediately after placement (Fig. 2). These regions comprised the vast majority of the perimeter of the SW implants placed into 3.75 mm drilling sites (Fig. 2a), and the outer aspects of the threads of the Unitite implants placed into the 3.75 (recommended, Fig. 2b) and both Unitite and SW implants placed in 4.0 mm (overdrilling, Fig. 2c,d) drilling sites.

At two weeks, the SW implants placed into the 3.75 mm drilling sites presented necrotic spots and bone remodeling sites (Fig. 2a) along the perimeter of the implant/bone contact especially in regions in close proximity with the implant inner diameter. At 4 weeks in vivo, the SW implants placed into the 3.75 mm drilling sites presented initial bone formation in the void space between threads that originated from remodeling of the necrotic spots, and remodeling sites were observed at bone regions in proximity with the thread tips (Fig. 2e).

For both Unitite groups and the SW implant placed in the 4.0 mm drilling sites, where a mismatch occurred between the implant inner diameter and the larger drilling site diameter (forming healing chambers), bone healing followed an intramembranous-type healing mode (Fig. 2c,d,g,h). At the 2 week time point, these implant groups presented spots of necrotic bone/ remodeling areas with bone resorption, and a chamber filled with osteogenic tissue between the implant inner diameter and the drilled wall along with newly formed woven bone (Fig. 2c,d). Primary engagement by the threads outer region without extensive necrotic bone areas was observed. At 4 weeks, the healing chamber regions presented higher amounts of newly formed woven bone, and regions where primary engagement between implant and bone occurred presented newly formed bone partially filling void regions where bone remodeling occurred (Fig. 2g,h).

**Figure 3:**
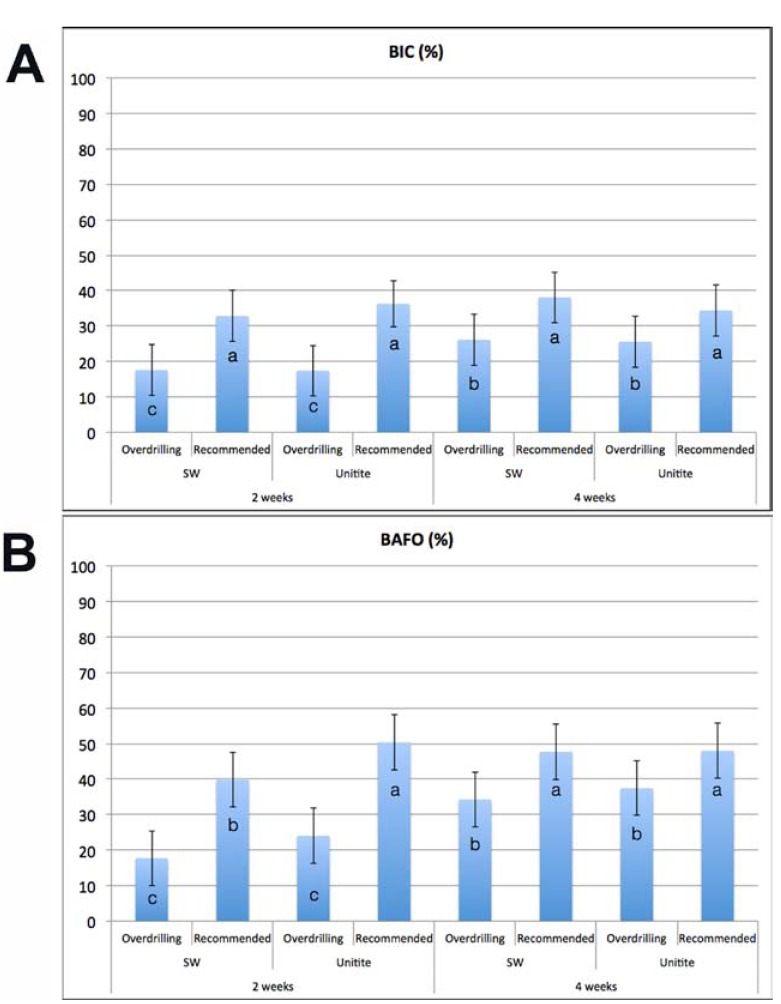
(a) Bone-to-implant contact (BIC) values for the two different implants placed under the recommended and the over drilling instrumentation protocols at 2 and 4 weeks. (b) Bone area fraction occupancy (BAFO) values for the two different implants placed under the recommended and the over drilling instrumentation protocols at 2 and 4 weeks. The error bars represent the 95% confidence interval and letters represent statistically homogeneous groups.

Statistical assessment of overall BIC as collapsed over implant design did not show significant differences between implant design groups (*p*=0.92, Fig. 3a). Within the different implantation times and drilling techniques, no significant differences were observed between implant designs. A significant increase in BIC was observed from 2 weeks to 4 weeks implantation time for the both implant designs placed with the over drilling technique (*p*<0.03). When both implants were placed in the 3.75 mm drilling sites, no significant differences were detected in BIC over time (*p*>0.32) (Fig. 3a).

A significant effect (*p*=0.044) of implant design was detected for BAFO measurements, where the Unitite implant presented higher values compared to the SW implants (Fig. 3b). An overall significant increase in BAFO was observed as a function of time in vivo (*p*<0.01). A significant increase in BAFO as a function of time was observed for all groups (all *p*<0.02) except for the Unitite implant design placed in 3.75 mm drilling sites (*p*>0.40), group that presented the highest BAFO levels at both evaluation times. At 2 weeks, the Unitite implant design presented higher values at both drilling techniques (statistically significant for the recommended 3.75 mm drilling sites, *p*<0.03). At 4 weeks, no significant differences in BAFO were observed between implant designs for each drilling technique (*p*>0.35) (Fig. 3b).

## Discussion

The present study evaluated two different implant designs placed into two different drilling dimensions, one recommended by the manufacturer and another drilling scheme where a larger final drill outer diameter was utilized. Since the implant designs selected were from the same implant manufacturer and presented the same body diameter and overall shape, the surgical drills were exactly the same. Both implants, as per the manufacturer, have the exact same indications and placement sequence. In general, the overall histometric results obtained in the present study were not surprising since BIC and BAFO were larger for the recommended drilling technique relative to the over drilling for both implants. Not surprisingly, higher insertion torque values were observed for the smaller diameter instrumentation relative to the larger diameter instrumentation. However, osseointegration pathways and kinetics substantially varied between designs depending on the drilling scheme utilized.

The rationale for the selection of the two drilling schemes on the two selected implant designs is that recent experimental preclinical work has shown the feasibility of achieving primary stability of dental implants through engagement of the implant thread outer portions while allowing for the formation of void spaces between implant and bone immediately after placement (healing chambers) (12). Since no bone resorption occurs in healing chambers and healing at those regions take place in an intramembranous-like rapid woven bone formation (23), such rapid bone growth may compensate for the implant stability loss due to compression regions where implant contacts bone for primary stability. From a clinical perspective, this phenomenon is of special interest in the rehabilitation with immediate/early functional loading of single implant crowns at regions of poor quality bone biomechanics, such as type IV bone. Theoretically, a higher amount of necrotic dieback and interfacial remodeling will take place, potentially decreasing implant stability over time until secondary stability is achieved through new bone formation between the implant surface and pristine bone. Although a clinical study has shown no differences in survival rates of implants placed in poor bone density under a regular or undersized drilling protocol, no information regarding prostheses loading schedule was provided, except that no immediate loading was performed (24). Therefore such information warrants further clinical investigation.

Previous studies have shown that healing chamber bone filling happens in tandem with the remodeling process at compression regions (8,14), and the results obtained in the present study further support this finding since irrespective of implant design and drilling scheme, regions compressed by the implant in close contact with bone underwent resorption and regions where void spaces were formed presented newly formed bone as early as at the 2 weeks time point. However, healing patterns substantially differed between designs and drilling techniques.

These differences in healing pattern were more remarkable between implants for the recommended drilling technique, where healing chambers were formed for the Unitite implant (as per its design rationale - healing chamber formation under the recommended drilling dimension) and direct contact occurred for most of the SW implant perimeter with bone. Thus, while bone was being resorbed between the SW thread regions, new bone was filling the void spaces that were allowed by the Unitite implant geometry and associated drilling dimension. Therefore, substantial remodeling occurred around the SW implant leading new contact between bone and implant taking place at 4 weeks (after interfacial remodeling). On the other hand, the Unitite implant presented remodeling taking place to lesser extent at the outer thread regions while presenting new bone in intimate contact with the implant as early as 2 weeks in vivo at the healing chamber regions. Due to the healing pathway dictated by the interplay between implant and surgical drilling dimension, histomorphometric analysis showed significantly higher BAFO for the Unitite implant relative to the SW at 2 weeks in vivo. Such significant difference was not observed at 4 weeks as new bone replaced the necrotic and void areas around the SW implant placed in the recommended drilling sites at 2 weeks. It is worth noting that such BAFO difference at 2 weeks in vivo between the SW and Unitite implants provided enough statistical size effect to deem significantly higher BAFO values for the Unitite implant relative to the SW when drilling technique and time in vivo were collapsed.

No differences in BIC were detected between implant designs under the recommended drilling procedure at 2 and 4 weeks and these results were likely due to the substantially different healing dynamics between the two different implant designs. It must be noted that while BIC is an important histomorphometric indication of osseointegration, it by no means represents the overall implant in bone system biomechanical competence, especially in cases where different implant designs are directly compared (25,26).

Healing patterns were similar between the SW and Unitite implants when the over drilling scheme was employed since larger healing chamber size was allowed for the Unitite implant while a healing chamber was formed for the SW implant. No difference in BIC and BAFO was observed between implant designs The over drilling sties both presented significantly lower BIC and BAFO compared to their recommended drilling scheme counterparts at both times in vivo. However their increase over time was higher, once again suggesting alterations in osseointegration pathway and kinetics between the experimental groups.
